# Sensitivity of Diffusion-Weighted STEAM MRI and EPI-DWI to Infratentorial Ischemic Stroke

**DOI:** 10.1371/journal.pone.0161416

**Published:** 2016-08-16

**Authors:** Ahmed A. Khalil, Marc Hohenhaus, Claudia Kunze, Wolf Schmidt, Peter Brunecker, Kersten Villringer, Klaus-Dietmar Merboldt, Jens Frahm, Jochen B. Fiebach

**Affiliations:** 1 Center for Stroke Research Berlin, Charité – Universitätsmedizin Berlin, Berlin, Germany; 2 NeuroCure Cluster of Excellence, Charité – Universitätsmedizin Berlin, Berlin, Germany; 3 International Graduate Program Medical Neurosciences, Charité – Universitätsmedizin Berlin, Berlin, Germany; 4 Klinik für Neurochirurgie, Universitätsklinikum Freiburg, Freiburg im Breisgau, Germany; 5 Biomedizinische NMR Forschungs GmbH am Max-Planck-Institut für biophysikalische Chemie, Göttingen, Germany; University of Glasgow, UNITED KINGDOM

## Abstract

**Objectives:**

To assess the sensitivity of stimulated echo acquisition mode diffusion weighted imaging (STEAM-DWI) to ischemic stroke in comparison to echo-planar imaging diffusion weighted imaging (EPI-DWI) in the infratentorial compartment.

**Methods:**

Fifty-seven patients presenting with clinical features of infratentorial stroke underwent STEAM-DWI, high-resolution EPI-DWI (HR-DWI, 2.5 mm slice thickness) and low-resolution EPI-DWI (LR-DWI, 5 mm slice thickness). Four readers assessed the presence of ischemic lesions and artifacts. Agreement between sequences and interobserver agreement on the presence of ischemia were calculated. The sensitivities of the DWI sequences were calculated in 45 patients with a confirmed diagnosis of infratentorial stroke.

**Results:**

Median time from symptom onset to imaging was 24 hours. STEAM-DWI agreed with LR-DWI in 89.5% of cases (kappa = 0.72, p<0.0001) and with HR-DWI in 89.5% of cases (kappa = 0.68, p<0.0001). STEAM-DWI showed fewer intraparenchymal artifacts (1/57) than HR-DWI (44/57) and LR-DWI (41/57). Ischemia was visible in 87% of cases for LR-DWI, 93% of cases for HR-DWI, and 89% of cases for STEAM-DWI. Interobserver agreement was good for STEAM-DWI (kappa = 0.62, p<0.0001).

**Conclusions:**

Compared to the best currently available MR sequence for detecting ischemia (HR-DWI), STEAM-DWI shows fewer artifacts and a similar sensitivity to infratentorial stroke.

## Introduction

Diffusion-weighted imaging (DWI) has a sensitivity exceeding 90% for detecting acute ischemia [1,2] and is routinely used for this purpose in clinical practice. However, commonly used DWI sequences, which combine a pulsed gradient spin-echo sequence and an echo-planar imaging (EPI) readout, show anisotropic diffusion and susceptibility artifacts. Such artifacts are particularly prominent in the infratentorial compartment and can hamper the detection of ischemic lesions in this area. The presence of these artifacts in the brainstem and cerebellum, where infarcts tend to be small and often inconspicuous, complicates the diagnosis of stroke using EPI-based DWI sequences [3–5].

DWI acquired with a slice-thickness of 2.5 mm is better at detecting ischemic lesions than that acquired with the more commonly used 5 mm slice thickness [6], most likely due to the higher spatial resolution. Therefore, despite taking longer to acquire and having a potentially lower signal-to-noise ratio, thin-slice DWI is considered the best available imaging tool in clinical practice for diagnosing cerebral ischemia. However, both these sequences are acquired with an EPI readout and therefore suffer from susceptibility artifacts. Diffusion-weighted images acquired using stimulated echo acquisition mode (STEAM-DWI) sequences are not affected by these artifacts [7–9], which makes them promising alternatives to conventional DWI sequences in infratentorial stroke. However, despite their potential advantages, the utility of STEAM-DWI sequences for diagnosing ischemic stroke has not yet been assessed.

In this study, we assessed the sensitivity to ischemia and the presence of intraparenchymal artifacts in three different DWI protocols (two EPI protocols with differing slice-thicknesses and one STEAM sequence). We hypothesized that STEAM-DWI would show fewer artifacts than both DWI protocols acquired using thin (2.5 mm) and thick (5 mm) slices and that the sensitivity of STEAM-DWI for detecting ischemia would be similar to conventional EPI-based DWI sequences.

## Materials and Methods

### Study design

This was a retrospective analysis of a prospective, single-center observational study (the 1000Plus study, ClinicalTrials.gov Identifier NCT00715533, full study protocol available here [10]), which received approval from the local ethics committee (Charité Ethikkommission, Ethikausschuss 4 am Campus Benjamin Franklin, Charitéplatz 1, 10177 Berlin; Institutional Review Board number EA4/026/08). All patients gave written informed consent.

### Participants

**Fig 1:**
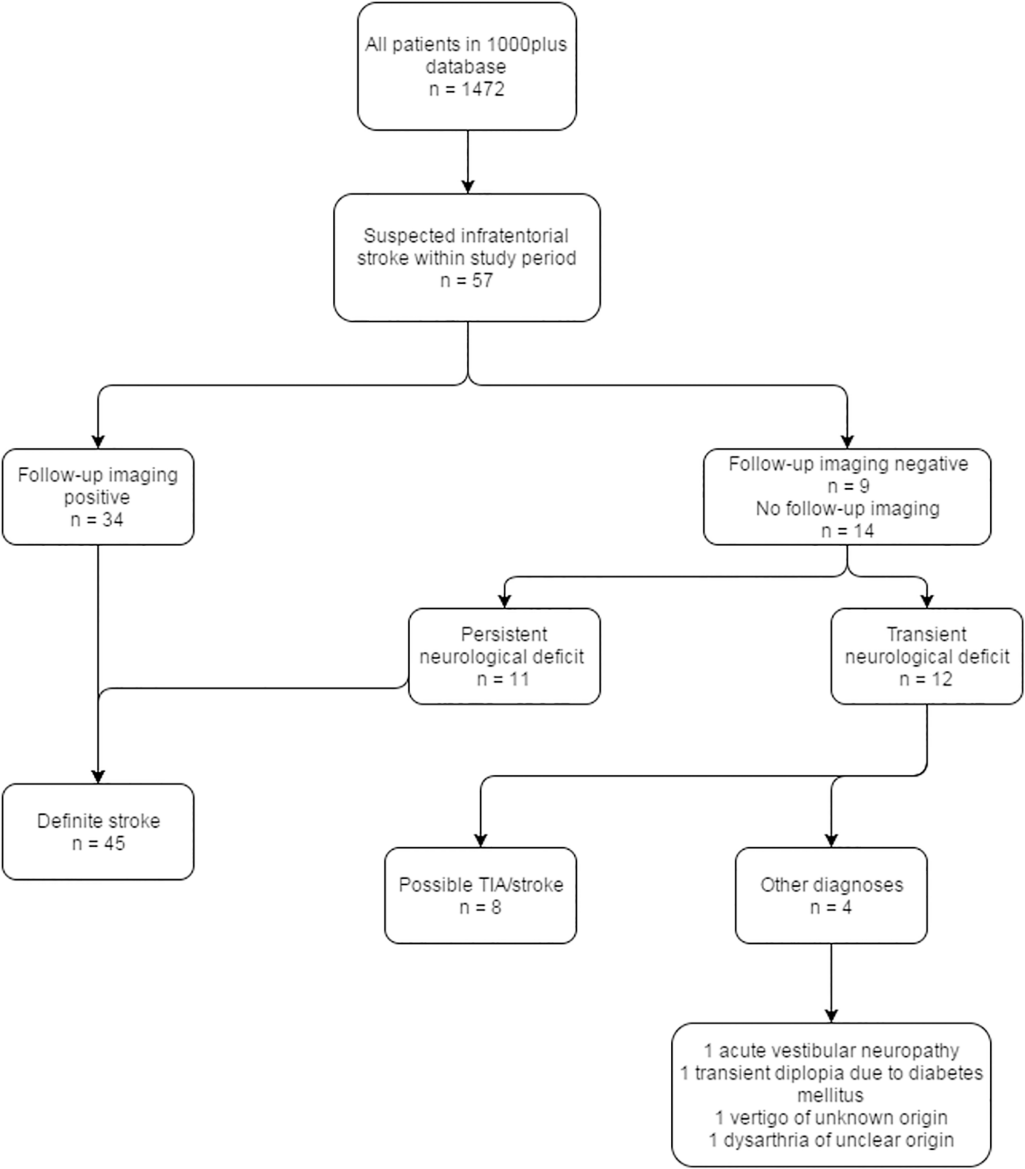
Flowchart of study participants.

Patients presenting to the emergency department who were clinically suspected of having an infratentorial stroke and who had no contraindications to MRI, were consecutively recruited between March 2010 and October 2011 at the Charité Campus Benjamin Franklin hospital in Berlin, Germany. Fig 1 shows a flowchart of the participants in this study.

### Test methods

MR images were acquired on a Siemens Tim Trio 3T scanner with a 12-channel head coil.

All patients received a thick-slice DWI sequence (referred to henceforth as low-resolution DWI, LR-DWI), a thin-slice DWI sequence (referred to henceforth as high-resolution DWI, HR-DWI), and a STEAM-DWI sequence in addition to a standard stroke imaging protocol, the details of which have been published by Hotter et al. [10]. All three DWI sequences were performed consecutively in the same scanning session without clinical intervention in between and their scanning parameters are shown in Table 1.

**Table 1:** Acquisition parameters for the DWI sequences.

Parameter	HR-DWI	LR-DWI	STEAM-DWI
**Slice thickness (mm)**	2.5	5	5
**Number of slices**	50	25	25
**In-plane resolution (mm)**	1.2 x 1.2	1.2 x 1.2	1.44 x 1.44
**Matrix**	192 x 192	192 x 192	160 x 120
**Repetition time (s)**	7.6	7.6	6.85
**Echo time (s)**	0.093	0.093	0.044
**Diffusion-encoding gradient directions**	6	6	6
**b-value (s/mm**^**2**^**)**	1000	1000	1000
**Number of averages**	2	2	5
**Acquisition time (s)**	133	91	330

Although HR-DWI is highly sensitive to ischemia [6], it nevertheless suffers from false-negatives [11] and we therefore considered it to be an unsuitable reference standard in this study. Instead, each patient’s final diagnosis was collected from the hospital records and discharge letters and was determined based on 1) clinical information, 2) follow-up MR imaging studies (FLAIR images performed at least one day and up to five days after the initial scan to confirm infarction and rule out alternative diagnoses), and 3) ancillary investigations including ECG, intra- and extracranial Doppler ultrasonography, and echocardiography. Patients received a final diagnosis of definite ischemic stroke if they had a persisting neurologic deficit at discharge or if they had imaging evidence of infratentorial infarction on follow-up MRI examinations. Similar to previous studies [4,5], this was used as the reference standard for all sensitivity calculations in this study.

### Image analysis

After completion of the recruitment phase, four readers who were blinded to all clinical information, including each subject’s final diagnosis, independently assessed the diffusion-weighted images. Two of them were senior, more experienced readers (a radiologist and a neuroradiologist with 7 and 14 years of stroke MRI experience respectively) and two were junior readers (a stroke researcher and a junior physician with 4 and 1.5 years of stroke MRI experience respectively). DWI images were randomly assigned with each reader assessing approximately half of the cases. Each image was assessed by both a senior and junior reader to calculate interobserver agreement.

**Fig 2:**
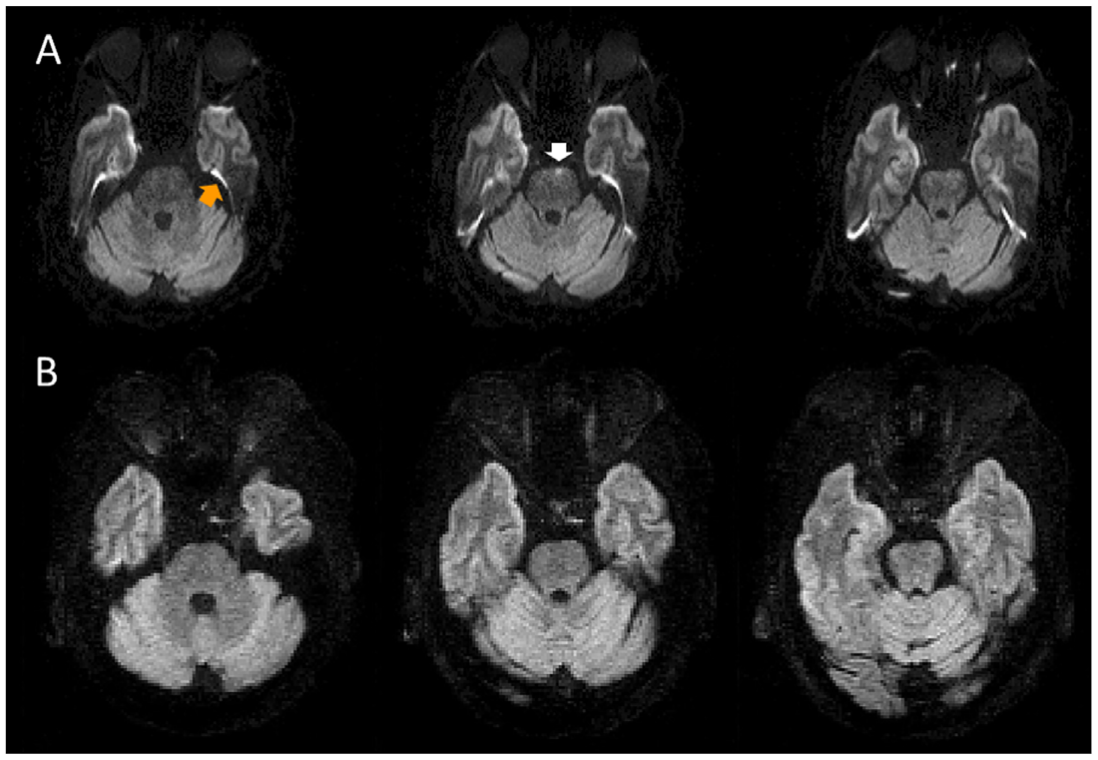
Artifacts on the DWI sequences. A) Example of artifacts on the high-resolution EPI-DWI (three consecutive slices shown). A typical susceptibility artifact is visible on the EPI-DWI (orange arrow)–note that such artifacts were ignored by the raters and are not the focus of this study. An intraparenchymal hyperintensity in the left pons (white arrow) is seen on a single slice. B) Neither of these artifacts is present on the STEAM-DWI.

The readers noted how many hyperintensities were visible on each diffusion-weighted image, the anatomical location of each hyperintensity, and whether it corresponded to an ischemic lesion or an artifact. Intraparenchymal hyperintensities that did not, in the readers’ experience, correspond to an infarction but would likely cause some diagnostic confusion in the setting of suspected infratentorial stroke were considered relevant artifacts (see Fig 2 for an illustrative example). Typical susceptibility artifacts seen in the interface between tissues (see Fig 2), particularly around the temporal lobe, were not considered relevant artifacts in this study because they do not clearly lie within the brain parenchyma and are unlikely to be considered ischemic in practice.

Image signal-to-noise ratios were calculated by dividing the mean signal intensity within the brain on a single slice by the standard deviation of the signal intensity in a region of interest (ROI) outside the brain on the B0 images. In addition, we calculated the lesion percentage contrast-to-noise ratios (CNR) for 10 of the patients using the trace DWI images. After registering the images to a template, a ROI was placed within the acute infarct. A second ROI was placed in healthy-appearing tissue in the contralateral hemisphere in the case of cerebellar stroke and on the opposite side in brainstem strokes. The mean signal intensity in the lesion (SL) and the mean signal in the healthy tissue (SH) were extracted and percentage CNR was calculated as follows: 100 x (SL–SH)/SH [1].

### Analysis

SPSS for Windows (Version 21; SPSS, Chicago, Ill) was used for statistical analysis.

Cohen’s kappa, overall raw agreement, and proportions of specific agreement [12] between the DWI sequences on the presence or absence of ischemic lesions (based on the senior readers’ judgement) were calculated and the intraclass correlation coefficient was used to evaluate the agreement on the number of ischemic lesions.

Sensitivity of each DWI sequence to ischemia was calculated, including 95% confidence intervals. The sensitivities of each sequence were compared using McNemar’s chi-square test.

In an exploratory analysis, we also calculated the sensitivity (and 95% confidence intervals) of each DWI sequence in patients presenting within 3 different time windows from stroke symptom onset to MR imaging (≤ 12 hours, > 12 ≤ 24 hours, and > 24 hours).

## Results

### Participants

A total of 57 patients were recruited for the study (see Fig 1); 39 males and 18 females with a mean age of 68 years (range = 44 to 92 years). Median NIHSS was 2 (range = 0 to 12) on admission and 1 (range = 0 to 9) at discharge. Median time from symptom onset to imaging was 24 hours (range = 2 to 217 hours, IQR = 47 hours).

### Presence and number of artifacts and ischemic lesions in the DWI sequences

At least one artifact was seen on 44/57 of the LR-DWI images (a total of 74 individual artifacts), 41/57 of the HR-DWI images (a total of 78 individual artifacts), and 1/57 of the STEAM-DWI images (a total of 1 artifact). Fig 2 shows examples of typical artifacts seen on the DWI sequences.

At least one hyperintensity due to ischemia was seen on 42/57 of the LR-DWI images (a total of 58 lesions), 46/57 of the HR-DWI images (a total of 71 lesions), and 44/57 of the STEAM-DWI images (a total of 60 lesions).

### Agreement between DWI sequences on the presence of ischemia

**Fig 3:**
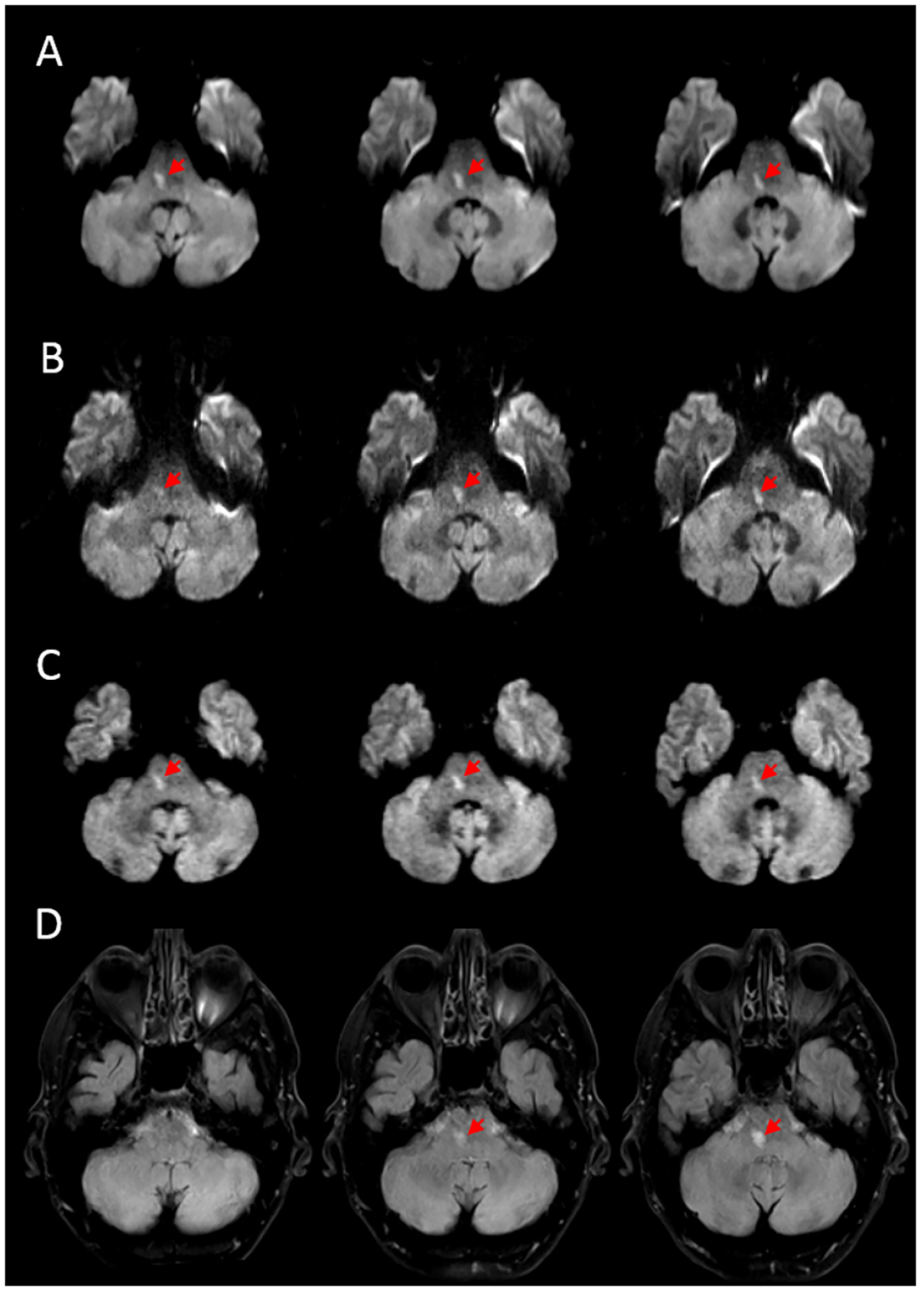
Representative example of agreement between DWI sequences for a patient studied 8 hours after onset of stroke symptoms. Both low- (A) and high-resolution (B) EPI-DWI show a hyperintensity in the right pons spanning three slices. C) STEAM-DWI also shows a hyperintensity in the right pons. D) FLAIR performed five days after baseline scans confirmed the presence of an infarct in the same location. Ischemic lesions are indicated by red arrows.

STEAM-DWI agreed with the LR-DWI in 51/57 (89.5%) of cases (kappa = 0.72, p<0.0001), positive percent agreement (PPA) was 93% (95% CI = 86.8–97.8%) and negative percent agreement (NPA) was 78.6% (95% CI = 57.1–93%). STEAM-DWI agreed with the HR-DWI in 51/57 (89.5%) of cases (kappa = 0.68, p<0.0001), PPA was 93.3% (95% CI = 87.4–97.9%) and NPA was 75% (95% CI = 50–91.7%). LR-DWI agreed with HR-DWI in 53/57 (93%) of cases (kappa = 0.80, p<0.0001), PPA was 95.5% (95% CI = 90.2–99%) and NPA was 84.6% (95% CI = 66.7–96.8%). Fig 3 shows a representative example of agreement between the DWI sequences. Table 2 shows a cross-tabulation of these results.

**Table 2:** Cross-tabulation of STEAM-DWI versus HR-DWI and LR-DWI in the whole sample.

		**HR-DWI**		**LR-DWI**	
		**Positive**	**Negative**	**Positive**	**Negative**
**STEAM-DWI**	**Positive**	42	2	40	4
	**Negative**	4	9	2	11

Results of stimulated echo acquisition mode DWI (STEAM-DWI) compared to high-resolution EPI-DWI (HR-DWI) and low-resolution EPI-DWI (LR-DWI) sequences on presence or absence of ischemic lesions in the entire sample, regardless of final diagnosis.

The intraclass correlation coefficients for the number of ischemic lesions detected was 0.84 (95% CI = 0.74 to 0.9) for LR-DWI versus STEAM-DWI, 0.63 (95% CI = 0.45 to 0.76) for STEAM-DWI versus HR-DWI, and 0.77 (95% CI = 0.62 to 0.86) for LR-DWI versus HR-DWI.

Agreement between the senior and junior readers on the presence of hyperintensities corresponding to ischemia was calculated for the LR-DWI (kappa = 0.67, p<0.0001), HR-DWI (kappa = 0.55, p<0.0001), and STEAM-DWI (kappa = 0.62, p<0.0001) sequences.

### Test results (sensitivity to ischemia)

Forty-five out of 57 patients had a confirmed final diagnosis of infratentorial infarction. Baseline imaging showed the presence of infarction in 39/45 (87%, 95% CI = 73.2–95%) of cases for LR-DWI, 42/45 (93%, 95% CI = 81.7–98.6%) of cases for HR-DWI, and 40/45 (89%, 95% CI = 76–96.3%) of cases for STEAM-DWI. The differences between sensitivities of STEAM-DWI and HR-DWI or STEAM-DWI and LR-DWI were not statistically significant (p = 0.623, p = 0.99, respectively).

**Fig 4:**
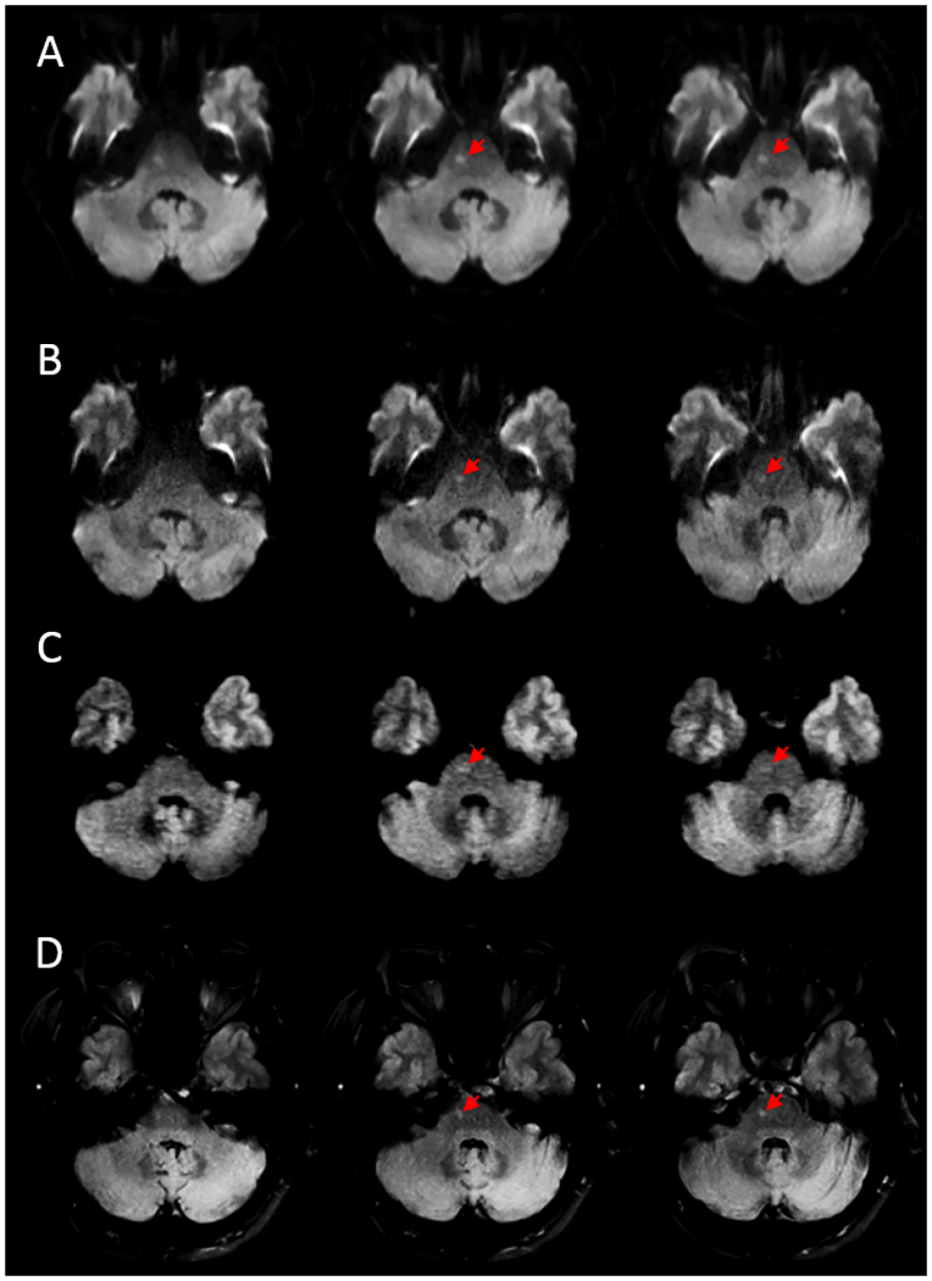
Example of disagreement between DWI sequences for a patient studied 19 hours after onset of stroke symptoms Both low- (A) and high-resolution (B) DWI show a hyperintensity in the right pons that was judged to be an artifact. C) The STEAM-DWI shows a hyperintensity in the right pons that was judged to be an ischemic lesion. D) FLAIR performed 5 days after baseline scans confirmed the presence of an infarct in the same location. Ischemic lesions are indicated by red arrows.

Of the false-negative DWI images, true infarcts (confirmed by follow-up imaging or consistent with the patient’s clinical deficit at hospital discharge) that were judged as artifacts by the readers were seen in 5 out of 6 of the LR-DWI, 2 out of 3 of the HR-DWI, and none of the STEAM images. In one patient, STEAM-DWI showed an ischemic lesion that was considered an artifact on the HR-DWI (see Fig 4).

**Fig 5:**
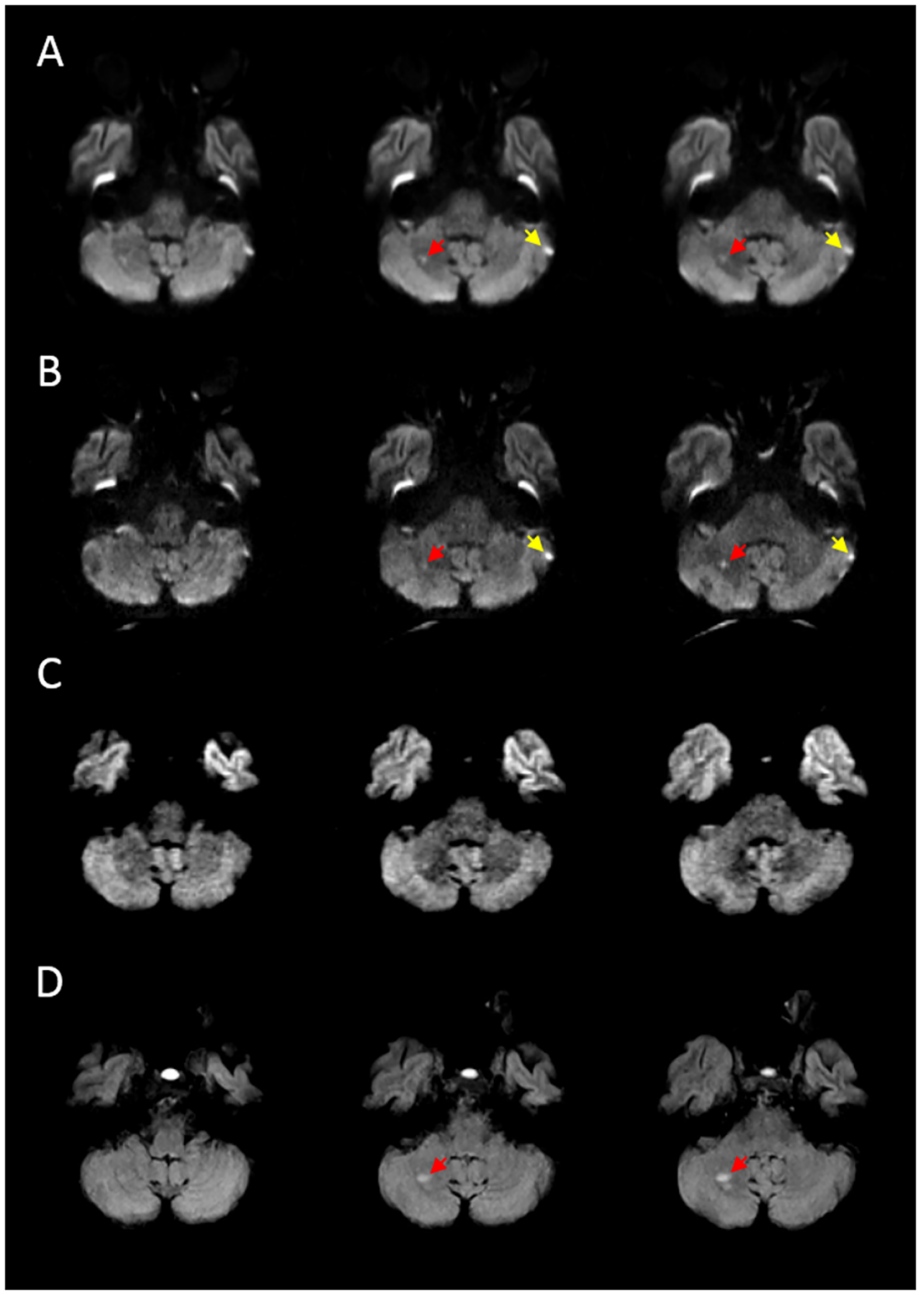
Example of disagreement between the DWI sequences. In this patient, MRI was performed 25 hours after the onset of stroke symptoms. Both low (A) and high-resolution (B) EPI-DWI show a hyperintensity (red arrows) in the right cerebellar lobe that was judged to be an ischemic lesion. Also note the hyperintensity in the periphery of the left cerebellar lobe (yellow arrows), which was judged to be an artifact. C) STEAM-DWI was judged as normal in this case. D) FLAIR performed 5 days after baseline scans confirmed the presence of an infarct in the same location.

Ischemic lesions were seen on the HR-DWI that were not detected on the STEAM-DWI sequence in three patients (see Fig 5). Table 3 shows a cross-tabulation of these results. Table 4 shows the sensitivity of each of the sequences to infarction stratified based on time from symptom onset to MR imaging.

**Table 3:** Cross-tabulation of STEAM-DWI versus HR-DWI and LR-DWI in patients with confirmed stroke.

		**HR-DWI**		**LR-DWI**	
		**Positive**	**Negative**	**Positive**	**Negative**
**STEAM-DWI**	**Positive**	39	1	37	3
	**Negative**	3	2	2	3

Results of stimulated echo acquisition mode DWI (STEAM-DWI) compared to high-resolution EPI-DWI (HR-DWI) and low-resolution EPI-DWI (LR-DWI) sequences on presence or absence of ischemic lesions in patients with a confirmed final diagnosis of infratentorial stroke.

**Table 4:** Sensitivity (and 95% CI) of DWI sequences to infarction (in patients with confirmed stroke) stratified by time from symptom onset to imaging.

	HR-DWI	LR-DWI	STEAM-DWI
**≤12 hours (n = 12)**	100% (73.5–100%)	100% (73.5–100%)	91.7% (61.5–99.8%)
**>12 ≤ 24 hours (n = 15)**	86.7% (59.5–98.3%)	80% (51.9–95.7%)	93.3% (68.1–99.8%)
**>24 hours (n = 18)**	94.4% (72.7–99.9%)	83.3% (58.6–96.4%)	83.3% (58.6–96.4%)

### Signal-to-noise and contrast-to-noise ratios

Image SNR (n = 53, mean +/- SD) was 78.7 +/- 16.0 for HR-DWI, 112.6 +/- 29.7 for LR-DWI, and 35.6 +/- 5.9 for STEAM-DWI. Lesion percentage CNR (n = 10, mean +/- SD) was 92.3% +/- 32.1% for HR-DWI, 73.7% +/- 27.4% for LR-DWI, and 43.5% +/- 12.5% for STEAM-DWI.

## Discussion

Diffusion-weighted imaging with stimulated echoes was used in some of the earliest studies investigating the detection of acute ischemia using MRI [13]. Since then, concerns about poor signal-to-noise ratios have overshadowed the potential advantages of this technique. To assess the practical implications of these concerns, we determined the agreement between two EPI-based DWI sequences and a STEAM-based DWI sequence and compared their sensitivities to infratentorial ischemic stroke. We found good agreement between STEAM-DWI and EPI-based DWI sequences and similar sensitivity to ischemia in these patients.

On EPI sequences, susceptibility artifacts in areas adjacent to the temporal lobes and brainstem can appear as signal hyperintensities on diffusion-weighted images and can easily be confused for small, localized brainstem or cerebellar infarctions (see Fig 2). Our results show that such artifacts are far less frequent in STEAM-DWI than in either of the EPI-DWI sequences. Conversely, very small areas of ischemia can be mistaken for artifacts on EPI-DWI sequences–this happened in several cases in our study and substantially contributed to the false negative rates of these sequences (see Fig 4). Despite having a similar false-negative rate, no true ischemic lesion was misjudged as being artifact in the STEAM-DWI sequence. These results reaffirm the notion that the presence of DWI artifacts is of practical importance because it can hinder stroke diagnosis. Thus, using a sequence lacking these artifacts, either instead of conventional EPI-DWI or as an add-on, may be particularly helpful when the diagnosis is unclear.

STEAM sequences have inherently lower signal-to-noise ratios than spin-echo EPI sequences [14] yet we found good overall agreement between STEAM-DWI and both HR-DWI and LR-DWI for detecting ischemia. Agreement between the DWI sequences by itself is not necessarily an adequate measure of the diagnostic performance of STEAM-DWI in a clinical setting because it does not rule out the possibility that both sequences being compared mistakenly diagnosed the cases. We therefore used a final diagnosis of infratentorial infarction, collected from follow-up imaging and other clinical data as performed in previous similar studies [4,5], as a reference standard to compare the actual sensitivities of the different DWI sequences. Our results show that despite having substantially lower contrast-to-noise ratios than EPI-DWI, the rate of detection of ischemic lesions was similar between the sequences.

We found a false-negative rate of 7% for HR-DWI and 13% for LR-DWI. Both these values are substantially lower than those reported by Oppenheim et al. (31% for HR-DWI) and Benameur et al. (56% for LR-DWI) in infratentorial stroke [4,6]. However these authors only studied patients presenting within 24 hours of symptom onset, when hyperintensities on DWI may be less conspicuous [15,16] and used lower magnetic field strengths than our current study. Overall sensitivity to ischemia detection was similar between STEAM-DWI and LR-DWI in our study and the sensitivity of STEAM-DWI was only slightly lower than that of HR-DWI. These results suggest that STEAM-DWI is able to dependably detect infratentorial ischemia in the majority (89%) of cases.

It is important to note that despite overall sensitivity to ischemia being similar, the STEAM-DWI sequence detected fewer (n = 60) ischemic lesions than HR-DWI (n = 71). The data suggest that this difference is likely due to slice thickness, because the number of lesions detected by STEAM-DWI was similar to LR-DWI (n = 58). Small, scattered lesions may appear to coalesce or partially disappear due to partial volume effects associated with low spatial resolution in the slice-select direction [17]. The number and pattern of ischemic lesions provides clues to stroke etiology [18]. Therefore, an important technical improvement to the STEAM-DWI sequence would be to acquire thinner slices, potentially improving its detection of multiple lesions.

Our study has some limitations worth mentioning. Artifacts in the STEAM-DWI sequence could have been classified as ischemic lesions based on the readers’ prior knowledge of the sequence’s properties, erroneously inflating its sensitivity. Because of this, we interpreted our data under the assumption that it is unlikely for a hyperintensity located in the same location as the final infarct to be an artifact mistaken for true ischemia. We therefore looked at the results of each sequence not only in terms of presence or absence of ischemic lesions, but also based on lesion location (although this was only possible for patients with follow-up imaging). Assessing the specificity of the different sequences could also have helped clarify the effect of this potential bias. However, this could not be done in our study because, unless a clear alternative diagnosis is established, the lack of imaging evidence of an infarction does not reliably exclude infratentorial stroke [19–21].

The use of a clinical reference standard itself is a potential limitation because there is inherent inter-observer variability in the diagnosis of stroke [22,23], which is closely related to clinicians’ experience [24]. Although our study was performed in a large center with considerable experience in managing stroke patients, the imperfect nature of the standard could have affected our sensitivity estimates. Our strict definition of a definite stroke also meant that sensitivity estimates could only be calculated based on 45 patients in our sample of 57. This modest sample size was the result of the prolongation of our routine stroke MR protocol by the addition of two sequences, which could not be justified in every case. However, our study was sufficiently powered to detect a difference of 15% or more between the sensitivities of EPI-DWI and STEAM-DWI [25]. This minimum detectable difference is markedly less than is expected based on the differences in SNR and CNR between the two sequences.

Our sample mostly consists of patients with subacute stroke but 12 patients with a confirmed diagnosis of infratentorial stroke were scanned within 12 hours of symptom onset. A single infarct was missed by the STEAM-DWI sequence in this subgroup, although this subanalysis was exploratory and should be assessed in larger prospective studies. Taking into account the fact that DWI lesions tend to be less conspicuous in the early hours following symptom onset [15], particularly in posterior circulation stroke [4], assessing the performance of STEAM-DWI in very early stroke is crucial. In our current study, performing such a time-consuming protocol (with three separate DWI scans) in an early time window could not be justified.

In this study, we attempted to compensate for the inherently low SNR of the STEAM-DWI sequence by increasing the number of averages, which led to longer acquisition times (5.5 minutes). However, attempts at shortening the total acquisition time of the STEAM-DWI sequence while maintaining or improving SNR can be achieved by using head coils with more channels that better support parallel imaging [26]. Further technical improvements may be achieved by adopting concepts recently developed for real-time MRI [27]. Such ideas include the use of radial encoding strategies and pronounced data undersampling [28] in conjunction with iterative image reconstruction by regularized nonlinear inversion [29,30]. This may allow studies in earlier time windows to be done.

## Conclusions

The results of our study show that STEAM-DWI suffers far less from artifacts mimicking ischemia than conventional EPI-DWI sequences. The sensitivity of STEAM-DWI to the presence of ischemia is similar to that of the best currently available MRI sequence used for this purpose, HR-DWI. With further technical development, STEAM-DWI may become a useful replacement or add-on to conventional DWI sequences in cases of suspected infratentorial stroke.

## Supporting Information

S1 Fig STandards for the Reporting of Diagnostic accuracy studies (STARD) checklist. (DOC)
